# The accuracy of chromosomal microarray testing for identification of embryonic mosaicism in human blastocysts

**DOI:** 10.1186/1755-8166-7-18

**Published:** 2014-02-28

**Authors:** Veronica Novik, Emily B Moulton, Michael E Sisson, Shagun L Shrestha, Khoa D Tran, Harvey J Stern, Brian D Mariani, Wayne S Stanley

**Affiliations:** 1 Genetics and IVF Institute, Fairfax, VA, USA

## Abstract

**Background:**

Most previous studies of chromosomal mosaicism in IVF embryos were performed by fluorescence *in situ* hybridization (FISH) methods. While there are reports implicating chromosome aneuploidy in implantation failure following transfer and pregnancy loss by spontaneous miscarriage, the significance of mosaicism for the developmental potential of growing embryos is unknown. However, the low prevalence of chromosomal mosaicism in chorionic villus sampling and amniotic fluid specimens suggests the presence of selection against mosaic embryos for implantation and early pregnancy. The absence of evidence for selective allocation of abnormal cells to the trophectoderm (TE) of mosaic blastocysts permits these cells to be a good proxy for embryonic mosaicism detection by chromosomal microarrays (CMA). The purpose of this study was to establish the limits of detection and the prevalence of chromosome mosaicism in day 5/6 human embryos using CMA with TE biopsies.

**Results:**

From reconstitution experiments we established log_2_ ratio thresholds for mosaicism detection. These studies indicated that chromosomal mosaicism at levels as low as between 25-37% can be consistently identified. Follow-up studies by FISH on non-transferred abnormal embryos confirmed the diagnostic accuracy of CMA testing. The number of cells in a TE biopsy can influence mosaicism detection.

**Conclusions:**

Chromosomal microarrays can detect mosaicism in TE biopsies when present at levels as low as between 25-37% and the prevalence of day 5/6 blastocysts which were mosaic and had no other abnormalities reached 15% among a cohort of 551 embryos examined. Validated protocols for establishing detection thresholds for mosaicism are important to reduce the likelihood of transferring abnormal embryos.

## Background

Chromosomal mosaicism has been associated with human morbidity but the phenotypic manifestations are dependent on mosaicism level, the chromosome involved, and tissue distribution. An accurate assessment of the prevalence of mosaicism in the newborn population is uncertain because of ascertainment bias toward abnormal clinical presentations. However, in prenatal specimens large studies indicate that mosaicism is found in 0.20-0.25% of amniotic fluid [1,2] and 0.8-2% of chorionic villus samples [3-6]. Chromosomal mosaicism is significantly higher in IVF created embryos than in other prenatal specimens. In a recent literature review and meta-analysis of human preimplantation embryo chromosomal mosaicism, van Echten-Arends et al. [7] reported that mosaicism is the most common chromosome constitution in spare IVF embryos and was found in 73% of all embryos. Of these embryos, 67% were at cleavage stage and most of the studies used FISH to determine chromosome constitution as well as spare embryos that were deemed unsuitable for transfer. Using chromosomal microarrays with cleavage stage embryos, Mertzamidou et al. [8] also found 71% of embryos to be mosaic. Most previous studies assessed mosaicism at cleavage stage from spare or arrested embryos. There is currently little data on chromosomal mosaicism derived from progressing day 5/6 hatching blastocysts.

Chromosomal microarrays have replaced FISH and comparative genomic hybridization (CGH) based methods for assessing aneuploidy in developing embryos [9,10] and aneuploidy has been implicated in implantation failure and pregnancy loss by spontaneous miscarriage following IVF embryo transfer [11,12]. The relatively low levels of mosaicism reported in prenatal specimens and the decreasing frequency of mosaicism with increased gestational age suggests the presence of selective pressure against embryos with high levels of mosaicism for ongoing pregnancy. Although mosaicism has been reported in embryos using CMAs with TE biopsies [13,14], the effects of this mosaicism on embryonic development, implantation, and pregnancy outcome is currently unknown and the degree to which a CMA result from a small sample of embryonic cells at day 5/6 accurately reflects embryonic mosaicism levels has not been adequately described. We report here on the limits of mosaicism detection using cell mixing experiments, the accuracy of mosaicism detection from FISH follow-up studies, and estimate the prevalence of mosaicism at the blastocyst stage of development.

## Results

### Reconstitution experiments and establishment of log_2_ ratios to detect chromosomal mosaicism

To mimic chromosomal gains and losses, mixing experiments with different ratios of amplified DNA from aneuploid and euploid cell lines as well as clinical samples were used as described in the Methods section. The sample size of five cells was chosen for SurePlex amplification because it is similar to the size of an average trophectoderm biopsy. Individual log_2_ ratios and average log_2_ ratios were determined for trisomy 13 and trisomy 21 samples at different levels of mosaicism (Additional file 1) and likewise for monosomy 6 and monosomy 22 (Additional file 2). A clear and definitive visualization of all hybridization signals above or below the 0 log_2_ ratio line, the first indicative sign of chromosomal mosaicism, was detected at levels as low as between 25-37.5% (a log_2_ ratio of +0.135) for gains and at 37-50% (an average log_2_ ratio of -0.153) for losses. The Additional files 3 and 4 provide illustrations of chromosomal aneuploidy at 0, 25, 37.5, 50, 75 and 100% for a trisomy (+13) and a monosomy (-6), respectively. Also of note is that the visual detection of chromosomal mosaicism is largely dependent on the quality of the starting material. Trophectoderm biopsy samples from degenerating embryos usually lead to a noisy pattern of array hybridization profiles which could obscure the detection of mosaicism. We blind-tested twelve mosaic and non-mosaic samples to further confirm our criteria – all samples were identified correctly (Additional file 5).

### FISH follow-up analysis of research embryos

The diagnostic accuracy of our CMA testing was assessed by FISH follow-up with non-transferred abnormal embryos. Only chromosomes identified to be aneuploid (non-mosaic or mosaic) by CMA were targeted by FISH probes. The results for embryos diagnosed as having at least one non-mosaic chromosome aneuploidy are shown in Table 1. For scoring purposes, an embryo FISH result was considered abnormal and concordant with the TE result when nuclei had the same abnormality identified by CMA or a complementary abnormality (e.g. +21 or -21) as could be found in mosaic embryos. Of the 26 embryos identified to be non-mosaic by CMA, FISH follow-up confirmed abnormalities in all of them (100%) but 50% of the embryos in this group were mosaic for at least one chromosome by FISH. The results for embryos diagnosed by CMA as having at least one mosaic chromosome aneuploidy are shown in Table 2. Of the 21 embryos in this group, 20 embryos were abnormal (95%). Nineteen embryos (90%) were confirmed as having at least one mosaic chromosome and one embryo was found by FISH to be non-mosaic aneuploid. For embryos with two or more mosaic chromosomes, FISH demonstrated that all these embryos contained some non-mosaic cells and thus did not represent aneuploid-aneuploid mosaic embryos. These results confirm the diagnostic accuracy of CMA testing for both non-mosaic and mosaic embryo aneuploidy categories and thus permit a realistic estimation of the prevalence of euploidy and aneuploidy in a larger cohort of day 5/6 blastocysts.

**Table 1:** Follow-up FISH data for embryos diagnosed with non-mosaic aneuploidy by CMA

**Specimen designation**	**TE cell #**	**CMA aneuploidy**^**a**^	**Log**_**2 **_**ratio**	**Abnormal nuclei by FISH (%)**^**b**^	**Non-mosaic**^**c**^	**Mosaic**^**d**^	**Embryo confirmed abnormal**^**e**^
1 NM	6	-X	-0.501	100	Yes	No	Yes
		-13	-0.461	100	Yes	No	
2 NM	3	+15	0.284	84	No	Yes	Yes
3 NM	2	-X	-0.721	37	No	Yes	Yes
		+Y	0.440	47	No	Yes	
		+21	0.369	47	No	Yes	
4 NM	2	-13	-0.409	21	No	Yes	Yes
		-18	-0.446	47	No	Yes	
5 NM	6	-22	-0.430	100	Yes	No	Yes
6 NM	5	-6	-0.487	100	Yes	No	Yes
7 NM	7	-14	-0.374	94	Yes	No	Yes
8 NM	7	+15	0.300	94	Yes	No	Yes
9 NM	2	-Y	-0.237	0	No	No	Yes
		+21	0.465	92	Yes	No	
10 NM	5	+15	0.317	97	Yes	No	Yes
11 NM	2	-15	-0.508	74	No	Yes	Yes
12 NM	4	-19	-0.382	91	Yes	No	Yes
13 NM	3	+5	0.294	80	No	Yes	Yes
		+14	0.249	83	No	Yes	
		-18	-0.529	100	Yes	No	
14 NM	3	-14	-0.555	73	No	Yes	Yes
15 NM	1	-22	-0.395	94	Yes	No	Yes
16 NM	4	-8	-0.642	97	Yes	No	Yes
		+11	0.336	97	Yes	No	
17 NM	3	-21	-0.432	73	No	Yes	Yes
18 NM	2	+9	0.375	94	Yes	No	Yes
19 NM	5	+16	0.253	86	No	Yes	Yes
20 NM	4	-22	-0.407	87	No	Yes	Yes
21 NM	3	-4	-0.440	12	No	Yes	Yes
		-10	-1.540	0	No	No	
22 NM	1-2	-X	-0.485	87	No	Yes	Yes
		+13	0.398	71	No	Yes	
23 NM	4	+8	0.288	94	Yes	No	Yes
		+22	0.268	97	Yes	No	
24 NM	8	-19	-0.417	80	No	Yes	Yes
25 NM	3	+22	+0.256	86	No	Yes	Yes
26 NM	3	-10	-0.561	94	Yes	No	Yes

^a^All chromosomes listed as abnormal in the embryos of this table were identified by BlueFuse software to be aneuploid.

^b^A nucleus is considered abnormal by FISH if there is a loss or gain of the chromosome identified by CMA to be aneuploid.

^c^A non-mosaic CMA result is considered concordant with FISH if ≥ 90% of follow-up embryo cells have a FISH signal pattern consistent with the CMA data.

^d^A mosaic CMA result is considered concordant with FISH if ≥ 10% and < 90% of follow-up embryo cells have a FISH signal pattern consistent with the CMA data.

^e^An embryo follow-up result is considered abnormal and concordant with an abnormal CMA result if at least one chromosome in an embryo identified by CMA as abnormal is confirmed by FISH.

**Table 2:** Follow-up FISH data for embryos diagnosed with mosaic aneuploidy by CMA

**Specimen designation**	**TE cell #**	**CMA aneuploidy**^**a**^	**Log**_**2 **_**ratio**	**Abnormal nuclei by FISH %**^**b**^	**Non-mosaic**^**c**^	**Mosaic**^**d**^	**Embryo confirmed abnormal**^**e**^
1 M	2	+14	0.193	97	Yes	No	Yes
2 M	4	+8	0.206	53	No	Yes	Yes
3 M	3	-1	-0.155	10	No	Yes	Yes
4 M	5	-4	-0.235	21	No	Yes	Yes
5 M	4	-19	-0.173	20	No	Yes	Yes
6 M	4	-4	-0.154	10	No	Yes	Yes
7 M	4	+5	0.220	27	No	Yes	Yes
		+10	0.133	6	No	No	
8 M	4	-8	-0.203	20	No	Yes	Yes
9 M	3	-14	-0.178	12	No	Yes	Yes
10 M	2	-9	-0.183	7	No	No	No
11 M	4	+15	0.230	63	No	Yes	Yes
		+17	0.164	20	No	Yes	
12 M	11	-8	-0.280	19	No	Yes	Yes
		-11	-0.141	28	No	Yes	
13 M	7	-8	-0.230	71	No	Yes	Yes
14 M	4	+2	0.211	40	No	Yes	Yes
		+8	0.181	26	No	Yes	
15 M	4	+22	0.136	16	No	Yes	Yes
16 M	4	+1	0.168	15	No	Yes	Yes
17 M	4	-18	-0.216	58	No	Yes	Yes
18 M	4	-8	-0.199	43	No	Yes	Yes
		+16	0.179	30	No	Yes	
19 M	2	-3	-0.146	23	No	Yes	Yes
		+X	0.159	19	No	Yes	
20 M	4	+19	0.159	23	No	Yes	Yes
		+X	0.149	26	No	Yes	
21 M	3-4	-19	-0.162	56	No	Yes	Yes

^a^Embryos listed in this table were identified by CMA to have at least one mosaic chromosome aneuploidy.

^b^A nucleus is considered abnormal by FISH if there is a loss or gain of the chromosome identified by CMA to be aneuploid.

^c^A non-mosaic CMA result is considered concordant with FISH if ≥ 90% of follow-up embryo cells have a FISH signal pattern consistent with the CMA data.

^d^A mosaic CMA result is considered concordant with FISH if ≥ 10% and < 90% of follow-up embryo cells have a FISH signal pattern consistent with the CMA data.

^e^An embryo follow-up result is considered abnormal and concordant with an abnormal CMA result if at least one chromosome in an embryo identified by CMA as abnormal is confirmed by FISH.

### Aneuploidy and mosaicism in day 5/6 embryos

From CMA analysis of 551 blastocysts obtained during IVF cycles at our clinic (Table 3), 52.5% were euploid and 47.5% were aneuploid. In the chromosomally aneuploid group, 40.5% were non-mosaic, 31.6% displayed chromosome mosaicism only (using the established criteria for chromosomal mosaicism described above), and the remaining abnormal embryos (27.9%) displayed both mosaic and non-mosaic forms of aneuploidy. Given that our follow-up studies indicated that about one half of CMA diagnosed non-mosaic embryos were mosaic, then the true number of embryos that are non-mosaic aneuploid is likely smaller and the mosaic only and mixed non-mosaic/mosaic aneuploid subgroups are likely larger. When this embryo cohort was analyzed by maternal age (Table 4), the percent of euploid embryos decreased with increased maternal age. There was also a direct relationship between increased maternal age and percent of non-mosaic aneuploid embryos; the ratio of non-mosaic aneuploid embryos to euploid embryos in the 25-34 age group was 0.24 but 1.67 in the age 40-42 group. The ratio of mosaic only embryos to euploid embryos was 0.26 in the youngest age group and 0.33 in the oldest group.

**Table 3:** Summary of CMA data on embryo mosaicism

**Total number of embryos analyzed**^**a**^	**Normal (%)**	**Abnormal, n = 262 (47.5%)**		
		**Aneuploid**^**b **^**(%)**	**Aneuploid**^**b **^**and mosaic**^**c **^**(%)**	**Mosaic**^**c **^**(%)**
551	289/551 (52.5)	106/262 (40.5)	73/262 (27.9)	83/262 (31.6)

^a^All age groups combined: the maternal age averaged across all blastocysts in cohort was 33.9. Embryos obtained from 120 PGD cycles (103 patients total). Donor eggs were used in 21 cycles; the maternal age for this group was calculated as 33.

^b^Embryo was called aneuploid if at least one chromosome was identified by BlueFuse software to be aneuploid at diagnosis.

^c^Embryo was called mosaic if at least one chromosome had a log_2_ ratio CMA result of ≥ +0.130 or ≤ -0.150 at diagnosis and a homogeneous deflection of all hybridization signals.

**Table 4:** Correlation between embryo euploidy/aneuploidy and maternal age

**Age group**	**Total number of embryos analyzed**	**Normal (%)**	**Abnormal: n varies according to the age group (%)**		
			**Aneuploid**^**a **^**(%)**	**Aneuploid**^**a **^**and mosaic**^**b **^**(%)**	**Mosaic**^**b **^**(%)**
25–34	338	202/338 (59.8)	48/136 (35.3)	35/136 (25.8)	53/136 (38.9)
35–39	163	75/163 (46)	38/88 (43.2)	24/88 (27.3)	26/88 (29.5)
40–42	50	12/50 (24)	20/38 (52.6)	14/38 (36.9)	4/38 (10.5)

^a^Embryo was called aneuploid when BlueFuse software called a chromosome(s) gain or loss.

^b^Embryo was called mosaic if at least one chromosome had a log_2_ ratio CMA result of ≥ +0.130 or ≤ -0.150 at diagnosis and a homogeneous deflection of all hybridization signals.

## Discussion

Chromosomal microarrays have documented aneuploidy and mosaicism to be common at the blastocyst stage of development [7,13-16]. While arrays can confidently identify non-mosaic aneuploidy, there is less certainty regarding the technical limits of detection of mosaicism especially when the biopsy cell number is limited and whole genome amplification is required. Because microarray data is dependent on array platform, the quality of DNA and software algorithms, it is important that individual laboratories develop their own protocols for assessing mosaicism. This has been recommended by both the American College of Medical Genetics and the European best practices guidelines for constitutional microarray testing [17,18]. As a prerequisite for assessing the accuracy of arrays for the identification of embryonic mosaicism, we first determined the limits of detection of mosaicism by performing reconstitution experiments using DNA mixed from normal and aneuploid samples and analyzing CMA results for mosaicism detection using BlueGnome’s 24sure array and data generated by BlueGnome’s BlueFuse Multi software.

In our experiments we attempted to identify and characterize the approximate lowest levels of mosaicism that could be confirmed from follow-up studies as biologically real. We interpreted the presence of whole chromosome mosaicism when a clear visual deflection of all hybridization signals above or below the log_2_ 0 ratio value was discernible for any chromosome. For chromosome gains, mosaicism as low as between 25-37% can be observed which corresponds to a log_2_ ratio of approximately +0.13 and for chromosome losses, mosaicism as low as between 37-50% can be observed which corresponds to a log_2_ ratio of approximately -0.15 (see Additional files 1, 2, 3 and 4). There was some variability around these cut-off levels, as expected, because BAC arrays have less reproducibility and more batch-to-batch variation when compared to oligonucleotide constructed arrays [19]. Variation in array lots and in whole genome DNA amplification with small samples can also influence log_2_ ratios. In addition, there are also likely to be minor chromosome specific log_2_ ratio differences between different individual chromosome trisomies and monosomies due to variation in the number of probes for each chromosome and their unique hybridization properties. The predicted log_2_ ratio for a chromosome gain is +0.58 [log_2_ (3/2)] and that for a chromosome loss is -1.0 [log_2_ (1/2)]. It might be expected, then, that deflection of hybridization signals below the log_2_ ratio 0 value would be more prominent for chromosome losses compared to gains and that mosaicism for losses would be relatively easier to identify than for gains. Others have demonstrated that copy number variation (CNV) duplications are obscured by lower levels of maternal cell contamination than for similarly sized CNV deletions [20]. In patient samples displaying both a chromosome gain(s) and loss(es), we usually find the deviation from the log_2_ 0 line is greater for chromosome losses than for chromosome gains (unpublished observations). In these situations, losses and gains were determined from the same pool of amplified DNA. However, in our reconstruction experiments where we did not detect lower levels of mosaicism for chromosome losses compared to chromosome gains, these results are likely to be an experimental artifact due to the combining of different pools of amplified DNA products where there could have been minor differences in the quality of the DNA prior to whole genome amplification. The results we obtained for chromosome gains are consistent with those of Mamas et al. [21] who used the same array platform and software but analyzed DNA extracted from mixed populations of aneuploid and normal cells to simulate mosaicism in PGD specimens. They noted a shift in the log_2_ ratio when mosaicism reached 25% and confidently interpreted the presence of mosaicism when it was present at the 50% level for chromosome gains. They did not present data for chromosome losses. Northrop et al. [14] combined cells, in graded proportions, from normal male and female cell lines to simulate mosaic blastocyst biopsies and then analyzed these mixed cell populations using a 262 K SNP microarray. Their results indicated that identification of monosomy X mosaicism (male cells) was detectable when > 25% of cells in the population were male. Scott et al. [22] mixed DNA samples extracted from cytogenetically normal and abnormal sources, without whole genome amplification, to determine the limits of mosaicism detection using a 44 K oligonucleotide array. Mosaicism as low as 10% for both gains and losses of whole chromosomes was detectable when data from dye-reversed replicates were combined but this detection limit rose to 20-30% mosaicism in the absence of dye-reversed replicates. While high resolution arrays may detect lower levels of mosaicism, the primary purpose of array testing in the PGD setting is to identify whole chromosome aneuploidy without the additional uncertainty associated with the interpretation of the clinical significance of small copy number variants. Therefore, with this clinical objective in mind and with the technical limitations imposed by small sample sizes and the requirement for whole genome amplification, embryo mosaicism of less than approximately 25% will not likely be detectable.

After establishing threshold levels for mosaicism identification, these criteria were applied to classify embryos as euploid, non-mosaic aneuploid, or mosaic aneuploid. Follow-up FISH studies of our abnormal embryos were evaluated to assess the diagnostic accuracy of CMA testing. In a series of 47 embryos with follow-up data, 26 embryos were considered to be non-mosaic by CMA. While FISH confirmed whole chromosome aneuploidy in all of the embryos (100%), 13 embryos in this group (50%) were mosaic by FISH for at least one chromosome. Among the possible reasons for this observation is unequal distribution of mosaic cells between the inner cell mass (ICM) and TE or the effect of sample size. Recent studies of cells derived from TE and ICM and analyzed by CMAs by Johnson et al. [23] and Northrup et al. [14] do not support the preferential segregation of aneuploid cells to the TE. These studies are also consistent with an earlier report by Evsikov and Verlinsky using FISH [24]. Because more embryonic cells are available for reanalysis by FISH compared to the limited number of cells in a TE biopsy, it is likely that some embryos diagnosed as non-mosaic aneuploid by CMA will display mosaicism by FISH due to sampling error. This observation has been reported by Fragouli et al. [13] when they compared CGH data and FISH reanalysis results. Therefore, we reported an embryo as confirmed abnormal if at least one chromosome in an embryo identified by CMA was also identified as abnormal (non-mosaic or mosaic) by FISH. These criteria are similar to that utilized by Gutierrez-Mateo et al. [25] in their study of embryo reanalysis by FISH. Another consideration regarding mosaic embryos is that embryos can be aneuploid-aneuploid or aneuploid-euploid. While the clinical significance of low levels of mosaicism is uncertain, most likely aneuploid-aneuploid mosaic embryos (no euploid cells) will not lead to viable live births. In our study all mosaic embryos were aneuploid-euploid.

If there is an equal distribution of cells within the embryo, a large biopsy will inherently better reflect the total embryonic population and will be less susceptible to sample bias compared to a small biopsy. Of the 13 embryos in the CMA non-mosaic category which by FISH follow-up were identified as mosaic, 10/13 (78%) of these embryos had three or less cells in the biopsy. Among 21 embryos identified as mosaic by CMA, 20 (95%) were confirmed as abnormal by FISH. Interestingly, one embryo in this group was non-mosaic by FISH. The single case with a discordant result had 7% of nuclei with an aneuploid FISH signal which was slightly below our threshold for scoring this embryo as abnormal. In this study we only attempted to confirm mosaicism by FISH for chromosomes identified by CMA to be abnormal. While it is possible that this approach underestimated the number of mosaic chromosomes present in embryos, this is not likely because in our preclinical validation studies we established high analytical sensitivity using CMA. No FISH follow-up studies were performed on embryos identified by CMA to be euploid because these embryos were generally not available for further study and because our preclinical validation experiments demonstrated high analytical specificity.

CMA testing of TE biopsies from unselected blastocyst populations demonstrates high levels of chromosomally abnormal embryos. In a recent randomized clinical trial, Yang et al. [16] reported aneuploidy in 56.6% of blastocysts from young, good prognosis patients. With aCGH(CMA) or CGH, Fragouli et al. [13] reported 42.3% blastocyst euploidy, 30% non-mosaic aneuploidy, and 32.4% mosaic aneuploidy. Using the mosaicism thresholds described above, we analyzed 551 day 5/6 embryos by CMA. Of these, 52.5% were diagnosed to be euploid and 47.5% to be chromosomally abnormal. In the abnormal embryo population, 40.5% were non-mosaic aneuploid, 31.6% mosaic only, and 27.9% of embryos displayed non-mosaic and mosaic aneuploidy together. However, based on our FISH follow-up studies that showed that about one half of CMA diagnosed non-mosaic embryos were, in fact, mosaic because of skewed sampling, then the size of the mosaic class of embryos would be expected to be larger. When the same CMA data were analyzed by age group, younger women (25-34 years old), as expected, had the highest percent of euploid embryos (59.8%) and older women (40-42 years old) had the lowest percent of euploid embryos (24%). The ratio of non-mosaic aneuploid embryos to euploid embryos was 0.24 in the youngest age group and 1.67 in the oldest age group. This observation is consistent with a maternal age effect for meiotic non-disjunction. The ratio of mosaic only embryos to euploid embryos was relatively constant among the different age groups: 0.26, 0.35, and 0.33 for the 25-34, 35-39, and 40-42 year old patients, respectively. While the number of embryos available for study for the 40-42 year old group was small and thus insufficient for statistical comparison, there does not appear to be striking differences between these groups for the mosaic only category. If larger studies confirm this observation, then mitotic non-disjunction leading to mosaicism would appear to be a random event unrelated to maternal age. With demonstrated maternal age effects on meiotic non-disjunction as well as egg cohort size, the practical significance of a steady rate of mitotic non-disjunction across age groups is that it can lead to a less complicated estimation of the number of normal blastocysts that can be expected given the number of fertilized eggs available and a women’s maternal age.

This study confirms that mosaicism is common in day 5/6 blastocysts and that CMA testing can identify a significant proportion of these embryos. FISH follow-up studies confirm that mosaicism suggested by CMA profiles is not likely to be due to technical artifacts of the arrays and thus the likelihood that normal embryos are misidentified and not transferred is reduced. Some abnormal mosaic embryos, however, may not be identified because the TE biopsy may not be representative of the embryo and it may yield only euploid cells. It is important, then, that laboratories perform pre-clinical mixing experiments to validate the performance characteristics of their array platform and to establish detection thresholds for mosaicism in order to reduce the chances of transferring abnormal embryos. Preliminary studies in our laboratory using next generation sequencing suggest that this approach can also be sensitive in the identification of blastocyst mosaicism.

## Conclusions

In this study we report the data from reconstitution experiments using DNA mixed from normal and aneuploidy specimens to define the limits of detection of mosaicism using CMA as well as the accuracy of aneuploidy and mosaic aneuploidy identification in blastocyst TE samples. Our findings underscore the challenges in the identification of embryos with low levels of chromosomal mosaicism and the importance of obtaining sufficient cells in the TE specimen to minimize the effects of skewed biopsy sampling.

## Methods

### Study design and materials

Experimental and laboratory studies were performed on karyotypically defined human fibroblast cell lines and human IVF embryos donated to quality assurance follow up. Patients’ CMA data was calculated based on analysis of 551 embryos obtained from 120 IVF cycles (103 patients total).

### Ovarian stimulation/IVF/TE biopsy

All embryos analyzed in this study were obtained during IVF cycles following ovarian stimulation by standard protocols as administered by the patients’ physicians. Intracytoplasmic sperm injection (ICSI) and day 3 assisted hatching procedures were routinely performed on all cases as required by our PGD protocol. The trophectoderm biopsy was done on day 5/6 hatching blastocysts, with an average biopsy size of around four-five cells based on microscopic estimation using a laser on the constricted area of herniating TE. A modification of Gardner’s blastocyst scoring system [26] was used for grading embryos that became hatching blastocysts on day 5 or day 6. In short, embryos were rated based on three criteria: (i) the degree of expansion – 6.2 to 6.6, (ii) the quality of inner cell mass – A to C, (iii) the quality of trophectoderm cells – X to Z. About 29% of all hatching blastocysts were scored as AX, ~25% were scored as AY, ~ 11% were score as BX, ~30% were scored as BY and ~5% were scored as AZ, CX, BZ.

### Reconstitution experiments

DNAs from aneuploid and euploid cell lines as well as clinical samples were mixed in different ratios (1:7 [12.5%], 1:3 [25%], 3:5 [37.5%], 1:1 [50%], 5:3 [62.5%], 3:1 [75%] and 7:1 [87.5%]) for reconstitution experiments. For chromosomal gains, trisomy 13, trisomy 21 and euploid male cell lines (GM00526, GM02067B and GM05386B, respectively; (Coriell Cell Repositories, USA) were used. DNA from five cells of each cell line was amplified using the SurePlex kit (BlueGnome, UK), mixed, and assayed by array comparative genomic hybridization (aCGH; BlueGnome). Reconstitution experiments to obtain the individual log_2_ ratios for trisomy 13 and trisomy 21 were independently repeated at least three times. For chromosomal losses, amplified DNA from patient specimens that were identified by CMA and confirmed by FISH to have full monosomy 6 and monosomy 22 were used. The trophectoderm biopsy samples from these sources contained five cells. For all reconstitution experiments, log_2_ ratios were calculated to express deviation of CMA profiles at each level of sample mixing. We interpreted the presence of mosaicism when all hybridization signals for a chromosome were uniform and visually separated above or below the 0 log_2_ ratio line; that is, when there was no scatter of individual hybridization signals or small groups of hybridization signals that closely approached or touched the 0 log_2_ ratio line (see Additional files 3 and 4). For a diagram of a reconstitution experiment please see the Additional file 6.

### CMA (aCGH)

The BlueGnome SurePlex and 24sure array kits were used for whole genome amplification (WGA) and subsequent labeling/hybridization of DNAs derived from fibroblast cells and TE specimens, following the manufacturer’s protocol (BlueGnome, UK). Slides were scanned using PowerScanner (Tecan, Switzerland) and TIFF images were analyzed and interpreted using log_2_ ratios calculated by BlueFuse Multi software (BlueGnome, UK). Embryos identified to be aneuploid (non-mosaic or mosaic) by CMA from TE specimens were reanalyzed, when available, by FISH to confirm the array diagnosis. The BlueFuse Multi software algorithm for making euploid and aneuploid calls with 24sure arrays is based on the median log_2_ ratio of each chromosome. A chromosome gain will be called when the median log_2_ ratio reaches +0.225 with 51% confidence in the call but will achieve 100% confidence when the median ratio is ≥ +0.35. A chromosome loss is called when the median log_2_ ratio is < -0.375 (51% confidence) and at 100% confidence when the median ratio is ≤ -0.6 (BlueGnome; personal communication). In addition to software algorithms, there are inter-experimental variables which also affect the ability to define the limits of detection of mosaicism. These include variability in array lots, biopsy size, and integrity of DNA in biopsied cells.

### Embryo follow-up studies

Blastocysts used for quality assurance follow-up studies were abnormal embryos donated for this purpose following an informed consent process. Establishing a legal disposition for every embryo created by IVF is mandatory. The purpose and importance of embryo follow-up studies is presented by a genetic counselor at the first PGD consult and detailed in patient take-home PGD materials. After getting this information, each patient must sign a written consent for embryos disposition – embryos may be frozen, discarded or donated to research. Patients are aware these decisions are entirely voluntary and that they will not directly benefit from them. The embryo donation research consent includes permission to publish aggregate data. The Genetics and IVF Institute (GIVF) Institutional Review Board (IRB) is registered with the United States Department of Health and Human Services (HHS) and adheres to their guidelines for the protection of human subjects. The research described in this publication consisted of the analysis of existing data from donated specimens obtained by written informed consent for the purpose of continuous PGD quality assurance (QA) monitoring. It was determined by the Chairperson of the GIVF IRB that this study qualified as exempt research as promulgated by the U.S. Department of Health and Human Services because the materials already existed for QA purposes and the analysis of records presented no risk to patients.

For FISH studies, blastocysts were dissociated by first removing the zona pellucida with 1000 μs bursts from a laser (Hamilton Thorne Biosciences, USA) or with treatment with 0.1% pronase. Embryos were then placed in 50 μl of Ca/Mg-free medium with 5% HSA [QA Medium with HEPES (Sage)/human serum albumin (100 mg/ml)] at room temperature for 15 minutes. Embryos were transferred to a 2 μl droplet of hypotonic solution (50% Dulbecco’s phosphate buffered saline) on a glass slide for approximately 2 minutes. To the hypotonic solution was added 15 μl of lysis solution (0.01 N HCl, 0.3% Tween 20). After approximately 10 minutes, most cells will have separated from each other with the cell membrane lysed. The slides were dried at room temperature or on a 37°C slide warmer. Cells were fixed for 5 minutes in 50 ml of 2.5% buffered formalin phosphate solution (Dulbecco’s phosphate buffered saline/10% buffered formalin phosphate) before being rinsed in deionized water and dehydrated for 5 minutes each in a 70%, 90%, and 100% ethanol series. Slides were dried at room temperature. FISH probes (Abbott, USA) used in follow-up studies targeted chromosomes identified to be abnormal by CMA analysis. Because of reduced hybridization efficiencies with multiple cycles of FISH on the same cell, only 2-3 chromosomes were targeted in follow-up in embryos containing multiple chromosome aneuploidies identified by CMA. Probes and target DNA were co-denatured at 75°C for 5 minutes using a HYBrite instrument (Abbott) and then transferred to a humidified chamber at 37°C overnight. Following hybridization, slides were first washed for 2 minutes in 0.4X SSC (sodium chloride/sodium citrate) at 72-72°C and then in 2XSSC/0.1% Igepal at room temperature for 1 minute. Slides were then rinsed at room temperature in PN buffer (100 mM Na_2_HPO_4_ / 50 mM NaH_2_ PO_4_ / 0.1% Igepal). Cells were counterstained with DAPI (Abbott, USA) and approximately 25-35 nuclei with good signal intensity were scored per embryo using a fluorescence microscope (Olympus) and a 60X oil immersion objective. The interpretation of interphase FISH data is subject to technical errors due to overlapping, split, weak, or faded signals. From our experience (unpublished data) and descriptions by others [27], we estimate there is an approximate 10% total error rate in FISH scoring when 2-3 probes are applied simultaneously. Therefore, we categorized embryos as non-mosaic aneuploid when 90% or more cells were scored as aneuploid and embryos as mosaic when < 90% of nuclei but ≥ 10% of nuclei had an aneuploidy for any single chromosome.

With an approximately 10% FISH error rate, it is possible that some abnormal embryos categorized by FISH to be mosaic could, in fact, have been non-mosaic when FISH results were near our upper threshold. In this instance, however, FISH would still have confirmed an abnormality in the embryo. It is also possible that some embryos with very low levels of mosaicism by FISH could have been euploid. In our follow-up experiments, however, very few embryos had FISH scores close to our lower threshold and thus this would not have materially affected the general conclusion of our study. Our CMA studies were validated only for whole chromosome aneuploidies and therefore, FISH follow-up data from embryos suggestive of containing segmental aneuploidies alone or in combination with other abnormalities were excluded.

## Abbreviations

aCGH: array Comparative genomic hybridization; CMA: Chromosomal microarrays; ICSI: Intracytoplasmic sperm injection; IRB: Institutional review board; IVF: *In Vitro* fertilization; FISH: Fluorescence *In situ* hybridization; PGD: Preimplantation genetic diagnosis; TE: Trophectoderm.

## Competing interests

The authors declare that they have no competing interests.

## Authors’ contributions

VN was involved in acquisition, analysis and interpretation of data, drafting and revising the article. EBM performed embryo spreading and FISH follow-up studies and helped with editing the paper. MES performed embryo spreading and FISH follow-up studies and helped with editing the paper. SLS took part in reconstitution experiments. KDT helped with acquisition of data. HJS substantially contributed to conception and design, critically revised the article and participated in the final approval of the version to be published. BDM substantially contributed to conception and design, critically revised the article and participated in the final approval of the version to be published. WSS helped to draft and revise the final version of the paper, and substantially contributed to the experimental design, and data interpretation. All authors read and approved the final manuscript.

## Supplementary Material

Additional file 1 **Reconstitution Experiments – Chromosome Gains.** Individual log_2_ ratios and average log_2_ ratios were determined for trisomy 13 and trisomy 21 samples at different levels of mosaicism. Click here for file

Additional file 2 **Reconstitution Experiments – Chromosome Losses.** Individual log_2_ ratios and average log_2_ ratios were determined for monosomy 6 and monosomy 22 at different levels of mosaicism. Click here for file

Additional file 3 **Chromosomal microarray profiles of chromosomal aneuploidy for a trisomy (+13).** Images (A) - (F) showed different levels of aneuploidy in the experimental samples: at 0, 25, 37.5, 50, 75 and 100%, respectively. Click here for file

Additional file 4 **Chromosomal microarray profiles of chromosomal aneuploidy for a monosomy (-6).** Images (A) - (F) showed different levels of aneuploidy in the experimental samples: at 0, 25, 37.5, 50, 75 and 100%, respectively. Click here for file

Additional file 5 Blind-test results of mosaic and non-mosaic samples. Click here for file

Additional file 6 **Diagram of a reconstitution experiment.** The visual illustration of how reconstitution (mixing) experiments were performed in this study. Click here for file
