# Supplementary material for: The accuracy of chromosomal microarray testing for identification of embryonic mosaicism in human blastocysts
Source: Mol Cytogenet. 2014 Feb 28;7:18. doi: 10.1186/1755-8166-7-18 (PMC3944884; doi:10.1186/1755-8166-7-18)
Supplement: Additional file 1 — Reconstitution Experiments – Chromosome Gains. Individual log2 ratios and average log2 ratios were determined for trisomy 13 and trisomy 21 samples at different levels of mosaicism. [file 1755-8166-7-18-S1.docx]

**Additional File 1.** **Reconstitution Experiments – Chromosome Gains.**

| **Aneuploidy %** | **Log_2_ ratio^a^** | | | | **Average log_2_ ratio** | **Deflection of signals^c^**  **(+13/+21)** |
| --- | --- | --- | --- | --- | --- | --- |
|  | **+13** | **SD^b^** | **+21** | **SD^b^** |  |  |
| 0 | -0.014 | 0.009 | 0.044 | 0.003 | 0.029 | No/No |
| 12.5 | 0.057 | 0.034 | 0.116 | 0.035 | 0.087 | No/No |
| 25 | 0.092 | 0.008 | 0.165 | 0.059 | 0.128 | No/Yes |
| 37.5 | 0.140 | 0.021 | 0.187 | 0.054 | 0.164 | Yes/Yes |
| 50 | 0.176 | 0.012 | 0.223 | 0.028 | 0.2 | Yes/Yes |
| 62.5 | 0.231 | 0.010 | 0.279 | 0.039 | 0.255 | Yes/Yes |
| 75 | 0.231 | 0.036 | 0.337 | 0.070 | 0.284 | Yes/Yes |
| 87.5 | 0.277 | 0.010 | 0.399 | 0.127 | 0.338 | Yes/Yes |
| 100 | 0.359 | 0.031 | 0.381 | 0.121 | 0.370 | Yes/Yes |

^a^The reconstitution experiments to obtain the individual log_2_ ratios for trisomy 13 and 21 were independently repeated at least three times.

^b^Standard Deviations for three independent determinations.

^c^Clear visual deflection of most hybridization signals above the 0 log_2_ ratio line for chromosomes 13 and 21.
