# Supplementary material for: The accuracy of chromosomal microarray testing for identification of embryonic mosaicism in human blastocysts
Source: Mol Cytogenet. 2014 Feb 28;7:18. doi: 10.1186/1755-8166-7-18 (PMC3944884; doi:10.1186/1755-8166-7-18)
Supplement: Additional file 2 — Reconstitution Experiments – Chromosome Losses. Individual log2 ratios and average log2 ratios were determined for monosomy 6 and monosomy 22 at different levels of mosaicism. [file 1755-8166-7-18-S2.docx]

**Additional File 2**. **Reconstitution Experiments – Chromosome Losses.**

| **Aneuploidy %** | **Log_2_ ratio** | | **Average log_2_ ratio** | **Deflection of signals^a^**  **(-6/-22)** |
| --- | --- | --- | --- | --- |
|  | **-6** | **-22** |  |  |
| 0 | -0.007 | -0.006 | -0.006 | No/No |
| 12.5 | -0.061 | -0.057 | -0.059 | No/No |
| 25 | -0.107 | -0.081 | -0.094 | No/No |
| 37.5 | -0.085 | -0.153 | -0.119 | No/Yes |
| 50 | -0.154 | -0.239 | -0.196 | Yes/Yes |
| 62.5 | -0.198 | -0.324 | -0.261 | Yes/Yes |
| 75 | -0.259 | -0.438 | -0.348 | Yes/Yes |
| 87.5 | -0.304 | ̶ | -0.304 | Yes/N/A |
| 100 | -0.419 | -0.505 | -0.462 | Yes/Yes |

^a^Clear visual deflection of most hybridization signals below the 0 log_2_ ratio line for chromosomes 6 and 22.
