# Supplementary material for: The accuracy of chromosomal microarray testing for identification of embryonic mosaicism in human blastocysts
Source: Mol Cytogenet. 2014 Feb 28;7:18. doi: 10.1186/1755-8166-7-18 (PMC3944884; doi:10.1186/1755-8166-7-18)
Supplement: Additional file 5 — Blind-test results of mosaic and non-mosaic samples. [file 1755-8166-7-18-S5.docx]

**Additional File 5**: **Blind-Test Results of Mosaic and Non-Mosaic Samples**

| **Sample #** | **Abnormality** | **Aneuploidy**  **(%)** | **Expected Log_2_ ratio** | **Detected Log_2_ ratio** | **Abnormality Identified**  **(Yes/No)** |
| --- | --- | --- | --- | --- | --- |
| 1 | Mosaic for trisomy 13 | 62.5 | ≥0.135 | 0.179 | Yes |
| 2 | Mosaic for trisomy 13 | 50 | ≥0.135 | 0.138 | Yes |
| 3 | Mosaic for trisomy 18 | 62.5 | ≥0.135 | 0.167 | Yes |
| 4 | Mosaic for trisomy 21 | 50 | ≥0.135 | 0.144 | Yes |
| 5 | Mosaic for monosomy 16 | 37.5 | ≤-0.153 | -0.179 | Yes |
| 6 | Mosaic for monosomy 14 | 62.5 | ≤-0.153 | -0.410 | Yes |
| 7 | Mosaic for monosomy 18 | 50 | ≤-0.153 | -0.317 | Yes |
| 8 | None | 0 | <0.135  >-0.153 | <0.135  >-0.153 | No |
| 9 | Mosaic for monosomy 14 | 50 | ≤-0.153 | -0.320 | Yes |
| 10 | None | 0 | <0.135  >-0.153 | <0.135  >-0.153 | No |
| 11 | Trisomy 15 | 100 | ≥0.255 | 0.373 | Yes |
| 12 | Monosomy 16 | 100 | ≤-0.375 | -0.602 | Yes |
